# Microbiomics: Novel Biomarkers of Colorectal Cancer Diagnosis and Prognosis

**DOI:** 10.3390/diagnostics16111582

**Published:** 2026-05-22

**Authors:** Lielong Yang, Wenjian Meng, Tinghan Yang, Yuzhou Zhu, Ziqiang Wang

**Affiliations:** 1 Colorectal Cancer Center, West China Hospital, Sichuan University, Chengdu 610041, China; 2 West China School of Clinical Medicine, Sichuan University, Chengdu 610041, China

**Keywords:** colorectal cancer, gut microbiome, biomarker, *Fusobacterium nucleatum*, diagnosis, prognosis, immunotherapy

## Abstract

With colorectal cancer (CRC) accounting for over 1.9 million new cases and 930,000 deaths globally in 2020, there is a critical need for innovative indicators to forecast disease advancement and therapeutic outcomes. The gut microbiome has emerged as a fertile area for discovering such diagnostic and prognostic signals. This narrative review collected current evidence on intestinal microorganisms and their metabolic products as candidate markers for CRC control. Intestinal communities influence malignancy through diverse mechanisms, including metabolic shifts, immune modulation, inflammation, proliferation/apoptosis regulation, genotoxicity, and mucosal barrier disruption. Pathogenic species, such as *Fusobacterium nucleatum* and enterotoxigenic Bacteroides fragilis, facilitate tumorigenesis via FadA-mediated signaling and Th17/IL-17 responses. In contrast, beneficial taxa like *Faecalibacterium prausnitzii* and *Akkermansia muciniphila* provide protective effects through short chain fatty acid production. Macrophage phenotype physiological equilibrium is altered and inflammatory status fluctuates under the former. Metabolically, hydrogen sulfide damages mitochondrial DNA and secondary bile acids stimulate cellular proliferation. While 16S rRNA sequencing and shotgun metagenomics are established detection strategies, innovative platforms like organoids and gene arrays remain in the exploratory stage. Clinical data indicates that *F. nucleatum* aligns with advanced tumor stage, and its combined detection with colibactin-producing *E. coli* achieves high sensitivity for early-stage screening. Additionally, *A. muciniphila* levels can anticipate the efficacy of PD-1 blockade immunotherapy. Microbiota-derived tools represent a transformative direction in oncology. Future research must focus on standardizing protocols and validating multi-marker panels to enhance clinical translation.

## 1. Introduction

Newly diagnosed colorectal cancer (CRC) cases rose to over 150,000 and ranked third in both sexes in America, 2024 [1]. The predicted death number of CRC is about 53,000 in the USA and 153,000 in the European Union during 2024 [1,2]. CRC casts persistent heavy burden on both individual family expenditure and worldwide hygiene. One of the most powerful factors in prejudging the disease outcome is tumor stage. Most CRC developed from benign polyps over a long term of 10 to 15 years. Proper adenoma detection and excision do break the incidence of CRC [3]. In England, Duke A stage CRC reached a 5 year overall survival (OS) as high as 98%, with a gradual decrease to 85%, 63% and 7.5% for Duke B, C and D stage diseases [4]. This result was identical to another study which concluded stage III CRC 65–70% 5-year survival and a steep reduction in stage IV to 13–14% [5,6]. A large-scale study based on the Martinique Cancer Registry between 1993 and 2012 reported a median OS of 2.0 years for stage III to IV CRC, while stage I to II had not reached that outcome before the program was terminated [7]. Efficient testing is urgently needed to advance diagnosis and prognosis.

So far, the most widely accepted screening methods include colonoscopy and the fecal immunohistochemical test (FIT). Colonoscopy provides a magnified view and appended clarification based on the directly acquired sample [8]. Although colonoscopy is the golden standard test in CRC diagnosis, the trauma and high expense discourage some patients. Studies concluded FIT displayed a sensitivity of 74% to 94% and a specificity of 85% to 95% in the diagnosis of CRC [9,10]. There is still a large unknown area left for particular anticipation. For example, for patients undergoing a watch-and-wait procedure after their lesion showed complete remission with CT or colonoscopy, a novel screen test able to predict its progress instead of just waiting until, in some cases, relapse would definitely be more safe. Another occasion is therapeutic predication. These further demands call for more markers other than just 100% accurate colonoscopy alone. The circulating tumor DNA (ctDNA) test and tumor mutation burden (TMB) are both innovative and derivative tumor examination methods at this time.

About 100 trillion microorganisms reside in the human gut, of which Firmicutes takes the lead proportion of approximately 67% and Bacteroidetes the second of 21% [11,12]. Previous evidence supported their role in some tumors, such as oral and breast cancer and malignant melanoma [13,14,15,16]. Similarly, the microbiomic test has great potential in predicting CRC outcomes, therapy-related or not. Recent studies demonstrated that a high level of *Fusobacterium nucleatum* and *Bacteroides fragilis* indicates poor prognosis [17]. The former is also reported to closely relate to chemotherapy resistance and distant metastasis [18].

Based on the accumulating assays on microorganisms, we conducted this narrative review to summarize the existing evidence, their differences and limitations, and the expectations of them as CRC biomarkers and associative mechanisms.

## 2. The Roles Microbiomes Play in CRC Formation and Progression

Human gut microbiomes affect colorectal carcinogenesis and progression in intricate pathways. To summarize, we collected the information listed below: metabolism, immune, inflammation, proliferation or apoptosis, genetoxin and barrier.

### 2.1. Metabolism

Microbiomes take carbohydrates, proteins, lipid, water and other nutrition from intraluminal contents, produce substances they need and excrete metabolic waste. Some natural or metabolic intermediate sulfur-containing proteins (like disulfide bonds) discharge hydrogen sulfide along degradation under particular enzymes catalysis [19]. This process is half adjusted by intestinal microbes [20]. Hydrogen sulfide plays a dualistic role in gastrointestinal micro-ecology. It enables electric transmission in the mitochondrial electron transport chain in complex II [21]. However, its overexpression inactivated complex IV and inhibited the mitochondrial respiratory chain [22,23]. This respiratory disruption results in the depletion of adenosine triphosphate (ATP) and the accumulation of reactive oxygen (ROS) [24,25]. Furthermore, hydrogen sulfide exceeds mitochondrial permeability transition and triggers hepatocyte apoptosis [26]. An overload of ROS strain injures both mitochondrial DNA (mtDNA) and nuclear DNA, thereby contributing to the genetic instability observed in CRC [23,27]. Additionally, H_2_S suppresses short chain acyl-CoA dehydrogenase, which downregulates the β-oxidation of butyrate, the primary energy substrate for colonic epithelial cells [28]. Desulfovibrio, a Gram-negative obligate anaerobic bacillus within the Proteobacteria phylum and a characterized sulfate-reducing bacteria (SRB), generates H_2_S via membranous and soluble sulfite reductase [29]. Contrary to most other microbiomes in the bowel that utilize the assimilatory sulfate reduction pathway, Desulfovibrio operates through a dissimilatory path [30]. This process of synthesizing hydrogen sulfide is detrimental to colonocyte respiration. Following accumulated ROS stress interrupted DNA stability and acted as a genetoxin by compromising DNA stability [31]. Additionally, colonic epithelial tight junction was broken by Desulfovibrio by activating transcription factor Snail 1 and altering the cellular location of occludin [32].

Parallel to sulfur metabolism, the microbial transformation of bile acids significantly modulates the colonic microenvironment. Bile acid is a group of hepatocyte-synthesized cholesterol derivatives promoting lipid digestion and absorption. Primary bile acids (PCAs) include cholic and chenodeoxycholic acids. They are delivered into duodenum once combined with glycine or taurine. Some PCAs are modified right after secretion into secondary bile acids (SBAs). Deoxycholic acid (DCA), lithocholic acid (LCA), isoLCA, alloLCA and other derivatives comprise the SBA family [33]. Meanwhile, bile acids physiologically regulate glucolipid metabolism and enterohepatic circulation through Farnesoid X Receptor (FXR) and Takeda G Protein-Coupled Receptor 5 (TGR5). Their excessive accumulation exacerbates chronic inflammation, induces double-strand DNA breaks and triggers aberrant cellular proliferation [34]. A recent experiment elucidated that mice exposed to high levels of DCA suffered raised risk of colonic adenoma converting into adenocarcinoma [35]. DCA enhances arachidonic acid excretion and prompts its transformation into pro-angiogenic prostaglandin and ROS, injuring DNA and curbing the restoration process. It also augments cyclooxygenage 2 (COX-2) via stimulation of the epidermal growth factor receptor (EGFR), which further impels colonic carcinogenesis or distant metastasis [36].

DCA-mediated bowel dysbiosis promotes the expansion of opportunistic pathogens such as Shigella and Desulfovibri and reduces beneficial Bifidobacterium and Lactobacillus populations [37]. In high-fat-diet-fed Apc^−/+^ CRC rodent models, SBAs stimulated colonic cell stemness and tumorigenesis through FXR activation, which is driven in part by ROS-induced ribosomal instability [38]. Such diets typically extend the proportion of Clostridium species and increase the DCA level. The latter upregulates hepatic stellate cell caducity-related expression and pro-inflammatory phenotype, which finally advanced into hepatocellular carcinoma [39]. Similarly, Clostridium was revealed to induce CRC development by other independent studies [40,41]. A high-fat diet potentially promoted PCA-producing microorganisms, accelerating tumor formation and process [42]. SBAs impose pressure on digestive ecology in various aspects including immune, inflammatory and microbial composition. LCAs inhibit hepatic-infiltrated macrophage glycolysis and facilitate intracellular oxidative phosphorylation, reshaping macrophages from anti-tumor M1 into the immune-suppressive M2 phenotype [43]. This adaption is synergized by reinforced M2 recruitment from the activated mannose receptor in CRC [44]. M1 is more aggressive as an inflammatory biomarker maintaining immune clearance. M2-type tumor-associated macrophages (TAMs) oppositely establish an immune privilege. Pathological suppression of FXR disrupted intestinal rhythm, resulting in diminished secretory immunoglobulin A (sIgA) and elevated Enterotoxigenic *Bacteroides fragilis* (ETBF) colonization, ultimately causing colitis-associated CRC [45].

In contrast to the potentially deleterious effects associated with hydrogen sulfide and SBAs, short chain fatty acids (SCFAs) refer to a saturated fatty acid with a limited length of carbon chain less than six carbon atoms. Acetic acid, propionic acid and butyric acid are common SCFAs within the human body. Butyrate, in particular, acts as a critical ligand for peroxisome proliferator-activated receptor γ (PPAR-γ), which is activated by butyrate, facilitating the metabolic transition into β-oxidation. This pathway provides energy supply for colonic epithelium, and blocks some pathogenic colonization [46]. Over 90% of SCFAs are rapidly absorbed in the proximal colon [47], while only approximately 5% are later excreted in feces [48]. The progression of CRC is frequently characterized by a marked reduction in SCFA-synthesizing taxa, specifically within the *Bacteroides* and *Firmicutes* phyla. Experimental evidence suggests that SCFA-producing flora enrichment restrain the PI3K/AKT pathway, triggering apoptosis-related genes, and reduce tumor volume [47,49]. Those phenomena align with clinical data demonstrating significantly depleted butyrate levels in the colonic microenvironment of CRC patients [50]. According to animal and cellular experiments, SCFAs inhibited pro-inflammatory factors, TNF-α, IL-18, IL-1β, IL-6 and iNOS, while simultaneously raising anti-inflammatory IL-10, repolarizing macrophages into the M2 type [51,52]. At the epigenetic level, butyrate functions as a histone deacelylases (HDAC) inhibitor, enhancing the methylation of Lysine 9 on histone H3 (H3K9) and downregulating STAT6 to further facilitate M2 macrophages formation [53]. Collectively, SCFAs are a group of anti-inflammatory substances downsizing potential energy-associated damages.

**Figure 1:**
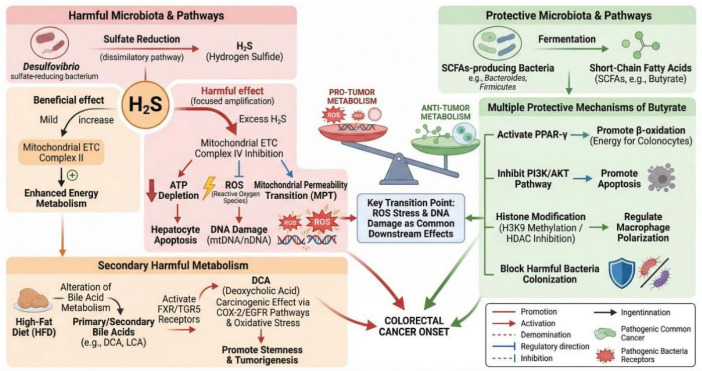
Gut microbiotic metabolites and their roles in CRC development. This picture illustrates the metabolic interplay between the intestinal microbiota and epithelium. Pro-tumorigenic signaling: Hydrogen sulfide induces mitochondrial dysfunction and oxidative DNA damage, while SBAs promote cellular stemness and chronic inflammation through FXR and TGR5 activation; Homeostatic and anti-tumorigenic signaling: SCFAs, primarily butyrate, serve as the principal energy substrate for the colonic epithelium and maintain the mucosal barrier. SCFAs exert potent anti-inflammatory effects by inhibiting histone deacetylases and inducing the differentiation of regulatory T cells to suppress tumor progression.

In addition to the above-discussed pathways, emerging evidence underscores the significance of other microbial metabolites including aromatic amino acids and lactate in CRC progression. Dysregulated aromatic amino acid synthesis disturbs mucosal barrier integrity. Colonic tissue samples collected from volunteers and patients supported a gradual elevation of some specified amino acids, L-alanine, glycine, L-valine, and myristic acid, from healthy controls, adenoma to CRC [54]. Knockout of the monocarboxylate transporter protein 2 gene has been shown to perturb populations of *Firmicutes* and *Bacillus* spp. Subsequent lactic acid excessive gathering drives macrophages into the M2 phenotype, thus promoting a pro-tumor microenvironment in respiratory and upper digestive malignant diseases [55,56]. In short, specified or common productions are core intermediate nodes for large quantities of microorganisms contributing to pro- or anti-CRC processes. Microbial metabolite effects and mechanisms are depicted in Table 1. Figure 1 illustrates microbial metabolites and product influences on CRC.

### 2.2. Immune

The gut microbiota modulates the host immune microenvironment through the secretion of various metabolic poisons or molecular modifications. *Fusobacterium nucleatum*, a Gram-negative staining obligate anaerobe colonizing in the human oral, gastrointestinal and urogenital tract, encodes a key virulence factor Fusobacterium adhesin A (FadA). It combines with T cell immune receptors containing immunoglobulin and tyrosine-based inhibitory motif (ITIM) domains, effectively suppressing T cells and natural killer (NK) cells via downstream signals [76]. The fusobacterial Gal-GalNAc-binding lectin Fap2 mediates *F. nucleatum* adherence to CRC membranous E-cadherin and propels cellular proliferation [77,78]. Concurrently, *F. nucleatum* induces the overexpression of toll-like receptor 4 (TLR4), actuating the failure of immunological surveillance and chemotherapeutic resistance [79]. *F. nucleatum* expands the population of CD11b^+^ myeloid-derived suppressor cells (MDSCs) including TAMs, dendritic cells (DCs), and granulocytes within the tumor immune microenvironment [80,81]. Notably, the abundance of *F. nucleatum* is positively correlated with CCL20 expression. The MiR-1322/CCL20 axis enhances the recruitment of TAMs and MDSCs via the NF-κB signal pathway, giving rise to M2 polarization markers, such as CD206, ARG1, IL-10 and TGF-β [82,83]. *F. nucleatum* reinforces suppressive T cells, TAMs, DCs and MDSCs, contributing to immune escape and promoting angiogenesis in CRC.

Bacteroides fragilis toxin (BFT), a zinc-dependent metalloproteinase synthesized by some detrimental *Bacteroides fragilis*, serves as a vital mediator in *B. fragilis*-induced colitis and CRC occurrence [84,85]. Studies found that recruited Th17 type regulatory T cells (Treg) and IL-17 are pivotal to the development of *B. fragilis*-associated CRC [86,87]. Distinct from the patterns observed in sporadic CRC, intensive MDSC infiltration were spotted within the BFT-constructed CRC microenvironment [88]. Specially, IL-17-aroused δγT cells attract CXCR2^+^ polymorphic nuclear innate myeloid cells and drive their differentiation into pro-tumor MDSCs [88,89]. Consequently, *B. fragilis* tends to establish an immune privilege microenvironment as soil for tumorigenesis centered on the regional enrichment of Th17 and high-level IL-17.

Beyond the representative bacterium discussed above, emerging evidence indicates immune regulation is a significant mechanism through which broader intestinal microorganisms exert influence on CRC. For instance, an experiment conducted in 2020 found that *Bacteroides* and *Bacillus faecalis* were linked with incremental Tregs infiltration in CRC [90]. A French team detected regional CD3^+^ T cell decline exhibited in Colibactin-Positive *Escherichia coli* (CoPEC)-colonized CRC, whether investigated in patients or murine animal models [91]. Compared with the control and adenoma group, colonic samples acquired from rats allocated to the colitis-associated CRC group exhibited a higher abundance of *Streptococcus gallolyticus*. They were shown to recruit MDSCs and lay a carcinogenetic microenvironment for CRC [92].

Conversely, probiotics exert protective influences through targeted immune intervention. Nontoxigenic *Bacteroides fragilis* (NTBF) secretes SCFAs that antagonize NLRP3-induced inflammatory signaling, thereby preventing excessive macrophage activation and finally suppressing colitis-associated CRC [52]. The synergistic interaction between NTBF and *Akkermansia muciniphila* expedites the development and maturation of DCs and upregulates IL-12 expression, which in turn enlarges the cytotoxic T cells population. This robust immune activation has been proven to multiply the therapeutic efficiency of immune checkpoint inhibitors (ICIs) in clinical practice [93,94]. Additionally, *B. adolescent* modulates the expression of GAS1 via the Wnt signal pathway, engaging CD143^+^ cancer-associated fibroblasts (CAFs) in an anti-tumor process [95]. Other *Bifidobacterium* strains further reinforce host defenses by increasing CD4^+^ and CD8^+^ T cells, and NK cells, while simultaneously optimizing the ratios of CD4^+^/Treg, CD8^+^/Treg, and effector CD8^+^/Treg to inhibit tumors [96]. To summarize, carcinogenetic flora facilitates a suppressive immune milieu while probiotics promote an anti-tumoral microenvironment.

### 2.3. Inflammation

Intestinal metabolic products play a fundamental role in the modulation of the colonic inflammatory landscape. Hydrogen sulfide not only breaks the mitochondrial respiratory chain, but also increases the risk of CRC via the inflammatory NF-κB pathway [23]. On the other hand, inflammatory recession on the basis of HDAC inhibition and G protein-coupled receptor expression enables SCFAs to minimize metabolic damages [97,98]. These metabolites exert potent negative regulation on pro-inflammatory factors, including TNF-α, IL-1β/6, and iNOS, while stimulating anti-inflammatory cytokine IL-10. Such molecular adaptions drive the polarization of macrophages toward the M2 phenotype and direct T cell differentiation to manage inflammation homeostasis [51,99].

The pro-carcinogenic inflammatory response associated with *Bacteroides fragilis* is primarily driven by its hallmark virulence factor, BFT. BFT increases the level of IL-17 and IL-23, boosts spermine oxidase (SPO) to produce ROS, disrupting regional inflammatory balance [49,100]. Subsequently promoted IL-17 and Th17 cells further trigger chemokines CXCL1, CXCL2, and CXCL5, appending secondary inflammatory cascade. Similarly, *S. gallolyticus* exacerbates CRC invasiveness by promoting the expansion of COX-2, IL-1 and IL-8 [101]. Investigations upon clinical samples collected from CRC or and other colorectal diseases support that the abundance of some protective species, such as *Faecalibacterium prausnitzii* and *A. muciniphila*, are significantly depleted relative to healthy controls. These taxa are thought to provide protective benefits through inflammatory restriction from Treg cells and butyrate [102,103].

In addition, emerging research indicates that colonic symbiotic fungi modulate the spleen tyrosine kinase (SYK)-caspase recruitment domain 9 (CARD9) signal axis, leading to inflammasome activation and increased IL-18 secretion. Mild uplift of inflammation heightened regional immune elimination and curbed CRC development [104]. However, extensive and long-term inflammatory infiltration will oppositely serve as a primary CRC driver.

### 2.4. Proliferation and Apoptosis

Cell cycle adaptation from intestinal microorganisms is put into effect under the alteration of some correlative intracellular signal pathways. NF-κB functions as a central hub in this regulatory network, consisting of a family of transcription factors that govern a broad spectrum of biological activities. Aberrant activation of NF-κB facilitates tumorigenesis by promoting cell survival and driving autonomous proliferation through the induction of key cell cycle regulators, including cyclin D1 and c-Myc [105,106]. Constitutive NF-κB signaling is a hallmark of various malignancies, such as lung and breast cancer [107,108]. Its role in CRC is closely intertwined with microbial dynamics. Specially, *F. nucleatum* has been observed to accelerate tumor progression in APC-deficient murine models by augmenting NF-κB signaling [82]. In *E. coli* enema-transplanted murine models, microbiota-stimulated secretion of cathepsin K (CTSK) via lipopolysaccharide triggers TLR4-dependent pathways and vitalizes CRC expansion and metastasis [109]. BFT similarly contributes to malignancy by promoting the Wnt, NF-κB, MAPK and STAT3 axis through Th17- and IL-17-mediated responses [72,86,110]. *Peptostreptococcus anaerobius* membranous signal domain PCWBR2 crosslinks with colorectal epithelial α2/β1 integrin, propelling an NF-κB and PI3K-Akt signal cascade to promote uncontrolled cellular multiplication [111].

Wnt/β-catenin is another significant proliferative pathway in CRC initiation and growth. When Wnt ligands interrupted degradation complex, stable β-catenin catabolic process came to a halt, stimulating downstream oncological targets including c-Myc and cyclin D1 and launching immoderate proliferation [112]. BFT increases the CRC proliferative rate via this pathway. The unique adhesive factor of *F. nucleatum*, FadA, expedites β-catenin by binding to epithelial E-cadherin to promote tumor formation [78]. In contrast, *Parvimonas micra* and *P. anaerobius* have been implicated in the inactivation of β-catenin, perturbing colonic epithelial renewal homeostasis. Such inordinate suppression of epithelial proliferation consequently brings about CRC [113]. On the contrary to these pro-proliferative influences, probiotics and their derivatives exert anti-tumor effects by inducing autophagy or apoptosis. SCFAs produced from members of *Bacteroides* and *Firmicutes* phyla effectively downregulate the PI3K/AKT signal pathway. The resulting pro-apoptosis effect facilitates the decrease in tumor mass [47]. Membranous exosomes secreted by NTBF have been found to start a non-canonical autophagy path and dispels established colitis-associated CRC [114]. Delicate homeostasis on proliferation or elimination is one of the central regulatory sites by which microorganisms impact CRC, as detailed in Table 2.

### 2.5. Genotoxin and Mucosal Barrier

Specific pathogenic microorganisms generated special bacteriogenic toxins categorized as genotoxins, which possess the capacity to induce structural DNA strand breaks. The most common representative one is colibactin, a polyketide-peptide genotoxin synthesized by *E. coli*. *E.coli* strains are phylogenetically classified into four groups: A, B1, B2, and D [118]. Colibactin is specifically encoded in the pks gene island of the B2 group [119,120]. That group is clinically correlated with inflammatory bowel disease (IBD) and tends to force out other group strains once civilized [121,122]. In rodent models of ulcerative colitis (UC), persistent inflammatory states induced pks expression, contributing to amplified mutation frequency and consolidated colitis-associated CRC [123,124]. Whole genome sequencing further has confirmed that colibactin-induced DNA double-strand breaks are predominantly enriched within adenine and thymine base pair enriched target motifs [125]. In addition to colibactin and other previously discussed toxins like BFT, *Campylobacter jejuni* synthesizes cytolethal distending toxin (CDT). The catalytic dissociation of DNA mediated by CDT is dependent on CDT b subunit since this part mutation reversed carcinogenesis [126].

**Figure 2:**
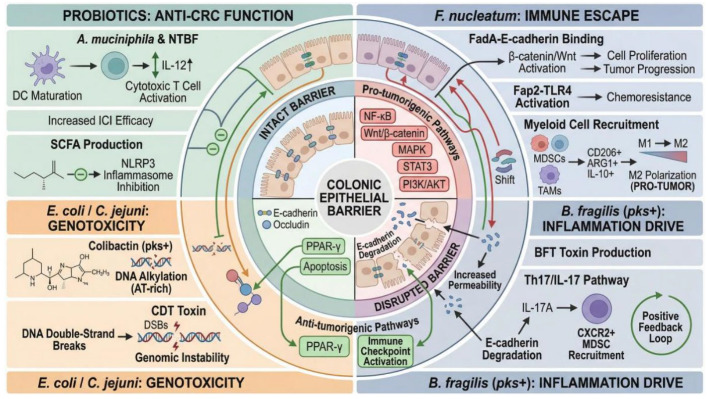
Mechanistic landscape of microbiomes and host interactions at the colonic epithelial barrier in CRC. This figure illustrates the divergent functional states of the colonic mucosal interface: homeostatic integrity and tumoral disruption. Green arrows represent for anti-tumor effects while red arrows for pro-tumor effects. Upper left section: Probiotic-mediated protection. *A. muciniphila* and NTBF reinforce anti-tumor immunity by facilitating DC maturation and IL-12-dependent cytotoxic T cell activation. SCFAs suppress tumorigenesis via inhibition of the NLRP3 inflammasome; Upper right section: Immune suppression and oncogenic signaling from *F. nucleatum*. FadA binds E-cadherin to activate the β-catenin/Wnt signaling axis, driving cellular proliferation. Fap2-mediated TLR4 activation contributes to chemoresistance. The recruitment of MDSCs and TAMs promotes an immunosuppressive M2 phenotype (CD206^+^, ARG1^+^, IL-10^+^); Lower right section: Chronic inflammation driven by ETBF. The secretion of BFT triggers a pro-carcinogenic Th17/IL-17 inflammatory cascade and recruits CXCR2^+^ MDSCs, ultimately inducing E-cadherin degradation and increased mucosal permeability; Lower left section: Microbial genotoxicity. Colibactin and CDT induce direct genomic instability via DNA alkylation at AT-rich regions and double-strand breaks, respectively.

The structural and functional integrity of the mucosal barrier serves as the primary defense mechanism protecting the intestinal epithelium against varieties of mechanical and biochemical injures. This barrier, composed of single layer of epithelial cells interconnected by collagen IV-based tight junctions, concurrently facilitates nutrition absorption and microbiomic isolation [127,128]. BFT impairs this barrier by inducing the proteolytic cleavage of E-cadherin and the subsequent dissolution of intracellular tight junctions [129]. In human-derived colonic cell lines, this process is characterized by the concomitant secretion of IL-8, whereas in rat colonoids, it is associated with a marked reduction in tight junction protein 1 (TJP1/ZO-1) [130,131]. *E. coli* operon afa-1 encodes non-classical adhesive factor other than pilus, assuring its invasion on colonocytes [132]. *F. nucleatum* utilizes FadA binding to E-cadherin, redeploying distribution of tight junction proteins to increase mucosal permeability [133]. *S. gallolyticus*, a Gram-positive non-motile bacteria streptococcus, exhibits a higher binding affinity for mucosal collagen I and IV than *S. bovis*, a property that facilitates the degradation of these extracellular matrix proteins and the subsequent attenuation of the epithelial barrier [134]. Approximately two thirds of patients presenting with invasive *S. gallolyticus* infections have concomitant CRC [135,136,137]. The complex interactions between these pathogenic microbes and the host mucosal environment are illustrated in Figure 2.

## 3. Contemporary Applications of Microbiological Markers in CRC Diagnosis or Prognosis

The intricate pathways through which the gut microbiome influences colorectal carcinogenesis, ranging from metabolic products and immune evasion to direct mucosal barrier disruption, establish a critical biological basis for their potential as clinical indicators. Building upon these mechanistic insights, current research seeks to translate the presence and activity of specific microbial taxa into actionable diagnostic and prognostic tools that can overcome the limitations of traditional markers. The technological platforms and genetic detective methods lay the basis for their clinical implementation.

### 3.1. Genetic Detective Methods and Carriers

On account of the specialized genetic sequences that encode respective microbial toxins, the quantification of these regions provides a comprehensive landscape of microflora construction and some potential information to predict the status of CRC. The most prevalent methodologies for fecal microbiological assessment include 16S rRNA testing and shotgun metagenomic sequencing [138,139]. The 16S rRNA gene, a component of the small bacterial ribosomal subunit, contains highly conserved sequences alongside nine hypermutable regions. This structure facilitates robust primer design and enables precise taxonomic identification across a diverse range of microbial taxa [140,141]. Compared to FIT with a fair sensitivity of 79% according to a meta-analysis, a multi-target DNA test ameliorates sensitivity to 92.3% accompanied by a marginal increase in false positive rate [142,143]. In contrast to amplicon-based methods, shotgun metagenomic sequencing circumvents targeted amplification by performing untargeted, high-throughput sequencing of the entire environmental DNA pool. By employing randomized DNA fragmentation, this approach allows for the reconstruction of metagenome-assembled genomes, enabling high-resolution identification at the species level and providing deep functional insights together. [144,145]. Data derived from these platforms support advanced investigations into drug tolerance, virulence factor profiles, biosynthetic gene clusters and other focused points [146]. To collect these clinical research efforts, several comprehensive metagenomic sequencing databases derived from both fecal and tissue samples have been established [41,73,147].

Organoids and gene arrays are novel test vectors under investigation. These technologies leverage the concept of genetic mutational signatures’ distinctive mutational patterns left in particular DNA districts after exposure to respective inducers. This property facilitates the application of organoid models in gastrointestinal flora systematic analysis [148]. Furthermore, the practical utility of serological antibody assays has been enhanced by their inherent advantages in cost efficiency and procedural accessibility [149,150]. As for the metabolites participating in CRC development, advanced analytical techniques like nuclear magnetic resonance (NMR) spectroscopy and various chromatographic methods assure their precise authentication and quantification. Table 3 summarizes methodologies in microbiological detection and their applications in the CRC field.

### 3.2. Microbial Biomarkers

Intestinal microbial composition undergoes significant flux during the pathological progression of CRC, spanning the transition from initial adenomatous lesions to malignant transformation and distant metastasis. The most predominant human colonic microbiota include *Firmicutes*, *Bacteroidetes*, and *Actinobacteria* phyla [154]. The Firmicutes-to-Bacteroidetes ratio, established as a dysbiosis index to reflect host gastrointestinal health status, has been verified as a relevant prognostic indicator. Studies illustrated that a higher dysbiosis index is correlated with prolonged survival outcomes in CRC patients [155,156]. Among specific taxa, *F. nucleatum* has become a focal point in this field with ample evidence supporting its predictive value on worse outcomes. Fecal and colonoscopy-obtained samples confirmed its higher propagation in CRC patients than healthy cohorts [157,158], with extensive enrichment within tumor tissues compared to adjacent normal mucosa [159]. A metagenomic analysis operated in three methods consistently identified the co-enrichment of *F. nucleatum* and *P. micra* as a hallmark of the CRC landscape [160]. A higher relative abundance of *F. nucleatum* indicated larger tumor volume, according to a clinical trial [161]. The FadA gene from colonic adenoma and adenocarcinoma samples expressed 10 to 100 times more than that of a healthy control [78], realizing the effective discrimination of adenomas from population screening [162]. In 2019, a large-scale study enrolling 606 patients again provided the consistent conclusion that *F. nucleatum* abundance was positively associated with CRC clinical stage progression from initial intramucosal cancer to advanced disease [41]. Significantly, a recent study found oral-harvested *F. nucleatum* DNA levels were relevant to CRC. The diagnostic curve highlighted its superiority over traditional carcinoembryonic antigen and carbohydrate antigen 199 (CA199) [163].

*E.coli* is another research node identified as a predicative indicator of early onset CRC [99]. Specifically, some tributaries of *E. coli* isolated from CRC patients demonstrate an overexpression of colibactin-encoding genes according to the Wassenaar research team [164]. In accordance with that, a case–control study discovered the gradual enrichment of colibactin-producing *E. coli* along with the whole development of colorectal adenocarcinoma. When united with *F. nucleatum*, the prediction on early CRC came up to a 63.1% specificity and 84.6% sensitivity and this is consistent with other studies [68,165]. Some other microbial species including *Clostridium perfringens*, *P. micra* and *Eggerthella. cordens* may serve as early predicative markers [54]. Similarly, *C. symbiosum* is regarded as a stage-related indicator [153], while members of the *Campylobacter* genus are documented as potential harbingers of elevated metastasis risk, as observed in a Chinese trial with initial I- to II-stage CRC patients [166].

For patients scheduled to receive immunotherapy, microbial biomarkers manifested considerable efficiency in potency anticipation. *A. muciniphila* is a Gram-negative staining obligate anaerobic bacteria connected with CRC. Studies proved a higher level of *A. muciniphila* led to a more active therapeutic response of programmed cell death protein 1 (PD-1) blockade. Thus CRC mice that received artificial complementary *A. muciniphila* exhibited improved PD-1 blocker curative effects [65]. This is attributed to the promotion of DC activation, Th1 polarization and augmented cytotoxic T cell recruitment [94].

Apart from evaluating disease status and potential chemotherapeutic resistance, microbial biomarkers serve as indicators of anatomical tumor localization, too. A multi-cohort analysis completed in 2025 proposed a CRC site-associated microorganic group, from which estimation on tumor location (right colon, left colon or rectum) attained an AUC of 82.92%. This investigation further documented a stepwise increase in α-diversity as the malignancy occurred more distally along the colorectal tract from right CRC, left CRC to RC [151]. The clinical applications and mechanistic roles of these diverse microbial markers are presented in Table 4.

The metabolic landscape of the colonic microenvironment undergoes significant remodeling during neoplastic growth, marked by the progressive enrichment of specific animo acids. Heightened concentrations of alanine, glutamine and glycine have been substantiated across various investigations as correlates of advancing disease stages [54,60,168,169,170]. Glycine functions as a vital metabolic precursor for the biosynthesis of nucleotides, lipids and proteins, which are essential components required to meet the high synthetic demands of proliferating tumor cells. The orchestration of glycine and serine within the one-carbon metabolism network fosters an immunosuppressive microenvironment, inducing an immune-suppressive microenvironment [171,172]. Complementing these amino acid signatures, other microbial-derived products, such as DCA and lactate, have been identified as reliable indicators for monitoring the progression of CRC [56,173].

## 4. Discussion

It was estimated that in 2020 over 1.9 million new CRC cases presented all over the world. A total of 930,000 deaths were attributed to CRC [174]. The survival outcome is inextricably linked to the stage at diagnosis. While traditional serum markers like carcino-embryonic antigen (CEA) and CA199 are widely utilized, their clinical utility is frequently compromised by poor specificity, as levels can be elevated by unrelated conditions such as smoking, IBD, liver diseases, pancreatitis and some other pathological alteration including liver cirrhosis, acute cholangitis, diabetes mellitus, endometriosis and bronchiectasis [175]. The missed diagnoses of some later-determined while not previously exhibited CRC cases lead to uncontrollable disease. Microbiomes have been evaluated in a number of malignant diseases including oral and breast cancer and malignant melanoma, etc., as a biomarker at different degrees of validity. They are at least equally effectual in CRC as they are located in close proximity.

The intestinal landscape contains a complex array of microorganisms that either drive or inhibit oncogenesis through distinct mechanisms. On the pathogenic side, *F. nucleatum* stands out as a primary driver of malignancy, utilizing its FadA adhesin to activate the β-catenin pathway and accelerate tumor proliferation. Furthermore, *F. nucleatum* is implicated in promoting chemotherapy resistance and is a reliable indicator of advanced tumor stages. Similarly, pks+ *E. coli* produces the genotoxin colibactin, which induces direct DNA double-strand breaks and genetic instability. ETBF further contributes to the pro-tumor environment by releasing BFT, a toxin that collapses the mucosal barrier and triggers a Th17-mediated inflammatory cascade.

Conversely, the microbiome also harbors protective members. *Faecalibacterium prausnitzii* and other fiber-fermenting bacteria synthesize SCFAs like butyrate, which serve as the primary energy source for colonocytes and exert anti-inflammatory effects. *A. muciniphila* plays a critical role in maintaining mucosal integrity and has been identified as a key predictor of positive responses to PD-1 blockade immunotherapy. NTBF also contributes to this protective equilibrium by suppressing inflammatory pathways and promoting immune elimination.

The findings suggest that the metabolites of these microbes are as significant as the microbes themselves. While excessive hydrogen sulfide and secondary bile acids like DCA promote DNA damage and cellular stemness, SCFAs act as a metabolic shield against carcinogenesis. Regarding detection strategies, 16S rRNA sequencing offers an economical option for large-scale screening at the genus level, but shotgun metagenomic sequencing is superior for high-resolution strain identification and functional metabolic analysis. Although innovative platforms like organoids and gene arrays show promise, they remain in an exploratory phase and require standardized operating procedures before broad clinical adoption.

Beyond basic diagnosis and prognosis, microbiological markers can reveal information about the entire development process of CRC and even indicate specific tumor locations. The latest studies have switched to investigating the therapeutic efficacy predication of leading modes represented by immunocheckpoint inhibitors. They have already attained periodical achievements. This topic calls for more clinical trials in the future, as currently available evidence relies heavily on animal experiments. Current evidence indicates that the obvious potential species have been analyzed extensively. Future efforts need to be made on further data treatment and reasonable filter to deduct dimensions and simplify the clinical application process. In terms of restricted predictive efficiency, suitable combinations might provide more beneficial selections. There is a to-be-established balance between simplification and efficacy. It might be a practical attempt to concentrate more on metabolites and their combinations as so far statistic process largely depends on microbiomic automated analysis which receives statistics submitted by microorganic detectors and panels. There may be a much simpler and more convenient method waiting to be discovered. Furthermore, though fungi, viruses and phages contributed less than germs, they should not be excluded from prospective investigations. The few studies on this topic have provided limited instructions. This, together with metabolite-based explorations, has great potential in the coming new era of CRC microbiomic biomarkers. It still remains to derive more potential useful microbiomic-related biomarkers and conduct clinical translation in response to the global CRC incidence accumulation in recent years. Ongoing and future lifestyle changes along with economic growth will only highlight the CRC-correlated predictive fields even more.

## 5. Conclusions

The gut microbiome and its associated metabolites represent a transformative frontier for the diagnosis and prognosis of CRC. As global cases and mortality rates continue to rise, there is a critical need to move beyond traditional serum markers like CEA and CA199, which often suffer from poor specificity. The intestinal landscape is a dynamic regulatory system where pathogenic species, such as *F. nucleatum*, ETBF, and pks+ *E. coli*, drive malignancy through FadA-mediated signaling, genotoxic colibactin production, and pro-tumorigenic inflammatory cascades. Conversely, beneficial taxa like *Faecalibacterium prausnitzii* and *A. muciniphila* maintain mucosal integrity and provide protective effects through the synthesis of SCFAs like butyrate.

The clinical integration of these microbiological indicators offers the potential to not only detect early-stage disease with high sensitivity but also to predict therapeutic efficacy for immunotherapies and identify tumor locations. However, to successfully translate these findings into routine oncology, future research must prioritize the standardization of detection protocols and the validation of multi-marker panels. Furthermore, as current evidence relies heavily on animal models, large-scale clinical trials are essential to confirm the utility of these biomarkers across diverse populations and evolving lifestyles. Ultimately, leveraging the gut’s microbial and metabolic signatures will facilitate personalized therapeutic strategies and significantly improve survival outcomes for CRC patients globally.

## Figures and Tables

**Table 1:** Microbial metabolites effects and mechanisms on CRC.

Substances and Toxins	Microbiomes	Effects and Mechanisms on CRC
Hydrogen sulfide	Sulfur-producing microorganisms: *Bilophila wadsworthia*, *Desulfovibrio*, *Pyramidobacter*, etc.	Genotoxicity: Direct DNA chains break and ROS elevation induced indirect damages [54]. Energy cutoff: Suppression on mitochondrial respiratory chain complex IV and butyrate application in energy supply of crypt cells [23,42]. Proliferation: Crypt epithelial cells proliferation ratio rise at about 50 percents level [42].
Deoxycholic acids	7α-Decarboxylating bacteria: *Clostridium scindens*, etc.	Angiogenesis: Promotion on colonic cytomembranous arachidonic acids excretion and COX-2 stimulation, facilitating neovascularization [42]. Propelling stem and invasiveness of CRC: FXR and TGR5 upregulation to endow stem. Deteriorating β-catenin pathway and adding aggressiveness to CRC [34,57]. DNA injures: ROS accumulation and DNA damages [44,49,58]. Cancer suppressor protein interruption: Proteasome activation and p53 protein degradation, selecting DNA injured cells survival to retain genetic instability [59]. Dysbiosis: Pathogen abundance augment and probiotics slash [58].
Lactate	General species	Macrophages repolarization: Reshaping TAMs into M2 phenotype [55]. Lift on tumor invasiveness: Inducing TAMs to secrete CCL17 and MDSCs infiltration [55].
Amino acid metabolites (alanine, glycine, etc.)	General species	Nutrition supply: Supporting biosynthetic precursors and energetic sources for exuberant tumor cells [54,60]. Synergism: Glycine cooperating with *P. anaerobius* on proliferation acceleration [54].
Butyrate	Fiber-fermenting bacteria including *Faecalibacterium prausnitzii*, Roseburia, *Clostridium butyricum*, Eubacterium, etc.	Predominant energetic origin of colonic epithelium [46]. Epigenetic regulation: Effectuating histone hyperacetylation as a histone deacetylase inhibitor [47,49,61]. Differentiation and apoptosis expedition. Immune mediated inflammation management: Hastening Tregs differentiation to restrain inflammation. Barrier restoration: Mucin synthetic enhancement and reconstruct damaged mucosal barrier [62].
Colibactin	pks^+^ *E. coli*	Genotoxin: Direct cleavage on host DNA double chains [57,63,64,65]. Genetic mutation: Leading to genetic instability and chromosomal aberration and leaving mutational signature within adenine-rich regions [66,67]. Cell aging: Urging cell aging and following secretion of HGF to spur CRC growth [63,68].
BFT	ETBF	Debonding epithelial barrier: Decomposition of E-cadherin, intra-cellular adhesion loss and permeability increase [57,65,69]. Proliferation: Secreting β-catenin to activate Wnt and MAPK signal pathways, raising c-Myc expression [50,70]. Immune suppression: Arousing IL-8 and TNF-α via NF-κB pathway, triggering Th17 and IL-17 mediated inflammation. Reinforcing immunosuppressive microenvironment by MDSCs recruitment [65,69,71,72]. Oxidative stress: stimulating SPO in ROS production [50,65].
FadA and LPS	*F. nucleatum*	Proliferation: β-catenin amplification by binding with E-cadherin and prompting carcinogenesis [62,73]. Chronic inflammation: Inflammation cascade initiation from combination of LPS and T cell expressed TLR4 [73,74]. Chemotherapeutic tolerance: 5-Fu therapeutic efficacy elimination through autophagy control [62,75].

**Table 2:** Proliferation- or apoptosis-associated signal pathways or targets.

Signal Pathways or Targets	Microbiomes	Launcher and Procedures
Wnt/β-catenin signal pathway	*F. Nucleatum*, ETBF, *S. gallolyticus*, *P. micra*, etc.	*F. nucleatum*: Combination of FadA and E-cadherin actuates β-catenin secretion and activation [68,115]. ETBF: Cleavage of E-cadherin from BFT enable β-catenin entrance into nucleus [72,73]. *S. Gallolyticus*: Nuclear β-catenin level elevation [70].
NF-κB signal pathway	*F. nucleatum*, *P. anaerobius*, ETBF, *E. faecalis*	*F. nucleatum*: TLR4 activation by LPS [50,57]. *P. anaerobius*: Membranous protein PCWBR2 adhesion with integrin [54,57,65]. ETBF: BFT expels inflammation by COX-2 and PGE2 in the downstream of NF-κB, facilitating CRC immune escape [57,115].
PI3K/Akt pathway	*P. anaerobius*	*P. anaerobius*: PCWBR2 binding with integrin α2/β1 starts PI3K/Akt pathway and promotes tumorigenesis [61,116]. SCFAs: Preventing PI3K/Akt pathway from excessive expression [47,58].
STAT3 pathway	ETBF, *F. nucleatum*, *Prevotella copri*	*F. nucleatum*: MDSCs infiltration through STAT3 pathway [50,57]. ETBF and *P. copri*: Selectively triggering STAT3 and Th17 cells differentiation, contributing to tumorigenesis [66,72]. STAT3 maintains survival and proliferative status in CRC cells [104].
TIGIT	*F. nucleatum*	*F. nucleatum* escapes from immune elimination under the binding of surface protein Fap2 and TIGIT expressed at T cells and NK cells [61].
FXR/TGR5	*Bacteroides*, *Clostridium*, etc.	*Bacteroides*, *Clostridium*, etc., transfer PCAs into SCAs. Later activation of TGR5 and FXR suppression consolidate stem and cause DNA damages [117].

**Table 3:** Methodologies in microbiological test and applications in CRC.

Approaches	Principles	Indicators	Characteristics	Advantages	Weaknesses	Significance
Metagenomic Sequencing (Shotgun Sequencing)	Random dissection on all genomes within feces and indiscriminate sequencing.	Specified bacteria abundance and functional genes.	High discrimination: Accurate quantification at bacterial strain level [73]. Functional analysis: Discovering genetic metabolic pathway alteration [40,139]. Position recognition: Distinguishing microbial proportions at different sites of colon and rectum [63,151].	Distinctions at bacterial strain level. Genetic information of various functional or metabolic pathways. High diagnostic precision for CRC with 0.84 AUC value [73]. Exemption from tumor stage leaded effects.	High volume of automatic analysis occupies huge hash rate [139]. Tissue sample is apt to lose efficacy when interrupted with host DNAs.	Decent identification on adenoma and early CRC. Improved sensitivity as united with FIT.
16S rRNA Sequencing	Conserved and hypervariable tRNA domains amplification and sequencing dependent of Fluorescence in Situ Hybridization (FISH).	Microorganic composition and class abundance.	Extensive classification: Flora diversity and community emerging [73]. Acceptable economic burden: Lower cost permits large-scale screening of 16S rRNA rather than metagenomic methods.	Mature test technology with existing substantial databases [139].	Distinguishing efficiency just reaches genus specificity [69]. No cellular function representation supported.	CRC risk: Diversity attenuation and *F. nucleatum* enrichment indicates incremental CRC possibility. Survival predication: Excessive colonization of *B. fragilis* and *F. nucleatum* are independent predictive factors for curtailed survival rate in CRC patients [115].
PCR-Based Virulence Gene Detection	Specified amplification of function gene related to toxicity or invasiveness.	Selected virulence factors like Colibactin and BFT.	Explicit target: Concentrating on pathogenic genes while not flora itself [67]. Timesaving: Standard procedure and rapid automatic process facilitate its utilization in practice [152].	High efficacy and convenience. Thorough translation into clinical medicine [153].	Constricted test fields with some potentially significant and undiscovered pathogens left [72,73].	Early prediction: *C. symbiosum* and Colibactin detection serve higher sensitivity in earlier CRC development [68,153]. Invasiveness prejudging: Clarifying highly invasive microbiomic subtypes [66,67]. United test scheme: Combination together with FIT attaches improved detective rate in CRC screening stage [153].
Metabolomic Analysis	Identification and quantification of microbial products within circulation, feces and other biological samples. NMR and mass spectrum technology are common methods.	Metabolites, such as DCA, sulfuretted hydrogen and butyrate.	Most direct and visualized function measurement. Suitable for extracting nutritional and dietary clues on CRC [42].	Functional regulation realizes upon genomic alterations is dependent on downstream substances. Metabolomics offered closest information of this part. Instantaneity: Other than subscribing clues among longer interval, metabolomics transmits instant circumstances within lower gastrointestinal tract.	Timely instability: Daily dietary patterns may attain a sudden change in a short time. Some temporary confounding factors cast unstable but obvious differences. Material fluctuation: Some products degrade or transform in poor control condition [42,73].	Stage evaluation: DCA, butyrate and alanine assist in judgment of distinct stage lesion from adenoma to cancer [35,50,54]. Barrier status assessment: SCFAs test for curative effects from probiotics or dietary administration. Butyrate level reflects epithelial barrier integrity [42,62].
Metatranscriptomics	Probe on transcription levels and directions from intestinal or fecal samples.	RNAs	Representation of virulent activity, not only existence [40,69]. Emphasis on more temporary effects than persistent ones [70].	Concentrating on functions ongoing, closer than DNAs. Containing more messages of recent coming adjustment than merely substances.	RNAs high instability calls for delicate preservation and brings about indeterminacy [69].	Not yet systematically put into clinical practice to date.

**Table 4:** CRC-associated microbiological markers and clinical applications.

Microbiomes	Indicative Fields	Mechanisms	Applications
*F. nucleatum*	Diagnostics, prognostics and therapeutic effect predication	Surface FadA ignite β-catenin signal pathway to accelerate proliferation. Inhibition on autophagy pathways significantly reduces chemotherapeutic response.	Adenoma and tumor diagnostic marker [63,69]. High abundance indicates shorten survival, cancer relapse and lymph node metastasis [63,115]. Reminding 5-Fu resistance [71,90].
pks^+^ *E. coli*	Diagnostics, classification and stages indication	Colibactin induces DNA double chains break and chromosome instability.	CoPEC is an early actuator of carcinogenesis [61,68]. Higher concentration in TNM III/IV stages mucosal tissue than earlier periods [63]. Tending to be positive in MSS CRC [63]. Enrichment in familial adenomatous polyposis [67].
ETBF	Diagnostics, classification and prognostics	Triggering STAT3 signal and lay pro-tumor inflammatory microenvironment by BFT release. Cleaving E-cadherin and initializing Wnt pathway and Th17 intermediated inflammation.	ETBF enrichment is an independent risk factor of CRC overall survival [115]. Remarkable relevance to CIMP, CpG gene island methylation [61]. Significant in early CRC screen [62,67]. Effective indicator of PD-1/PD-L1 immunotherapy [62].
*P. anaerobius*	Diagnostics and position marker	Combining membranous PCWBR2 with integrin. Instigating PI3K-Akt signal to expedite uncontrollable proliferation.	Climbing from adenoma to CRC as a potential early set biomarker [65,116]. Characteristic signature of rectal cancer [151].
*C*. *symbiosum*	Diagnostics	-	AUC value of advanced adenoma or early CRC surpasses that of *F. nucleatum* [153]. Multistep uprising from healthy state, adenoma, early cancer to advanced CRC [65,153].
*Faecalibacterium prausnitzii*	Diagnostics and prognostics	A source of butyrate.	Dramatic reduction along tumorigenesis [167]. Positively correlated with long-term cancer related and overall survival [115]. Postoperative abundance restoration prompts intestinal functional recovery [115].
*A. muciniphila*	Curative effect predication	Enhancing Th1 cells and renovating bowel mucosal integrity.	Ample colonization of *A. muciniphila* is positively relevant to treatment efficacy [61,63,65].
*S. gallolyticus*	Diagnostics	Upregulating IL-1, IL-8 and other inflammatory cytokines to build tumor microenvironment.	*S. gallolyticus* caused bacteremia declares latent CRC [69,70].

## Data Availability

No new data were created or analyzed in this study.

## Abbreviations

CRC, colorectal cancer; FIT, fecal immunohistochemical test; ROS, reactive oxygen; SRB, sulfate-reducing bacteria; PCAs, primary bile acids; SBAs, secondary bile acids; DCA, deoxycholic acid; LCA, lithocholic acid; FXR, Farnesoid X Receptor; TGR5, Takeda G Protein-Coupled Receptor 5; TAMs, tumor associated macrophages; ETBF, *Enterotoxigenic Bacteroides Fragilis*; SCFAs, short chain fatty acids; HDAC, histone deacelylases; FadA, Fusobacterium adhesin A; MDSCs, myeloid-derived suppressor cells; DCs, dendritic cells; BFT, bacteroides fragilis toxin; CoPEC, Colibactin-Positive *Escherichia Coli*; CAFs, cancer-associated fibroblasts; IBD, inflammatory bowel disease.
